# Effect of Phytosynthesized Selenium and Cerium Oxide Nanoparticles on Wheat (*Triticum aestivum* L.) against Stripe Rust Disease

**DOI:** 10.3390/molecules27238149

**Published:** 2022-11-23

**Authors:** Muhammad Shahbaz, Noor Fatima, Zia-ur-Rehman Mashwani, Abida Akram, Ehsan ul Haq, Asma Mehak, Fozia Abasi, Maryam Ajmal, Tayyaba Yousaf, Naveed Iqbal Raja, Hammad UlHassan, José Manuel Pérez de la Lastra

**Affiliations:** 1 Department of Botany, PMAS-Arid Agriculture University Rawalpindi, Rawalpindi 46000, Pakistan; 2 Department of Botany, Lahore College for Women University, Lahore 54000, Pakistan; 3 Department of Agronomy, PMAS-Arid Agriculture University, Rawalpindi 46000, Pakistan; 4 Department of Biology, Faculty of Sciences, PMAS-Arid Agriculture University, Rawalpindi 46000, Pakistan; 5 Biotechnology of Macromolecules Research Group, Instituto de Productos Naturales y Agrobiología (IPNA CSIC), 38206 San Cristóbal de la Laguna, Spain

**Keywords:** wheat, *Puccinia striformis*, nanobiotechnology, antioxidant activity, antifungal activity, SeNPs, CeONPs, green synthesis

## Abstract

In this study, selenium nanoparticles (SeNPs) and cerium oxide nanoparticles (CeONPs) were synthesized by using the extract of *Melia azedarach* leaves, and *Acorus calamusas* rhizomes, respectively, and investigated for the biological and sustainable control of yellow, or stripe rust, disease in wheat. The green synthesized NPs were characterized by UV-Visible spectroscopy, scanning electron microscopy (SEM), energy-dispersive X-ray (EDX), and X-ray diffraction (XRD). The SeNPs and CeONPs, with different concentrations (i.e., 10, 20, 30, and 40 mg/L), were exogenously applied to wheat infected with *Puccinia striformis.* SeNPs and CeONPs, at a concentration of 30 mg/L, were found to be the most suitable concentrations, which reduced the disease severity and enhanced the morphological (plant height, root length, shoot length, leaf length, and ear length), physiological (chlorophyll and membrane stability index), biochemical (proline, phenolics and flavonoids) and antioxidant (SOD and POD) parameters. The antioxidant activity of SeNPs and CeONPs was also measured. For this purpose, different concentrations (50, 100, 150, 200 and 400 ppm) of both SeNPs and CeONPs were used. The concentration of 400 ppm most promoted the DPPH, ABTS and reducing power activity of both SeNPs and CeONPs. This study is considered the first biocompatible approach to evaluate the potential of green synthesized SeNPs and CeONPs to improve the health of yellow, or stripe rust, infected wheat plants and to provide an effective management strategy to inhibit the growth of *Puccinia striformis*.

## 1. Introduction

Wheat (*Triticum aestivum*) is an important and staple food crop in Pakistan, contributing about 2.2% of the country’s total GDP [1]. However, a survey conducted for the period 2014–2019 showed a decline in wheat production from 2.2% to 0.5%, which is a serious concern for Pakistan’s food security [2]. Wheat consumption is predicted to increase by 60% by 2050, primarily in developing countries, to fulfill the food demands of growing populations [3]. This increased demand for wheat means that wheat diseases are a very serious issue, because wheat diseases are responsible for 12.5% of wheat yield loss annually all over the globe, and, among all these pathogens, the fungal pathogens are the chief disease-causing mediators in wheat [4]. Fungal diseases cause huge wheat losses, by damaging the physiological, biochemical, and antioxidant defense systems of wheat, ultimately resulting in low food quality and quantity [5].

Stripe, or yellow rust, is caused by the fungus *Puccinia striiformis* f. sp. Tritici. Striperust and is the most damaging wheat disease, affecting root growth, plant height, number of grains per spike, and, consequently, contributing to decreased dry matter, yield, and grain quality [6,7]. Seeds of plants affected by stripe rust have low vigor and, therefore, form poor seedlings after germination. Stripe rust can destroy the crop if the infection occurs very early [8]. The average wheat yield loss caused by stripe rust ranges from 25% to 80% worldwide [9].

Fungicide applications and resistant varieties can effectively combat stripe rust. However, they have negative impacts on crops over time, which ultimately harm ecosystems and human health. Additionally, *P. striiformis* constantly produces new virulent genes (pathogen variants) in the pathogen population, which results in diminution of host resistance [10]. Modern technologies can help to minimize the use of toxic agrochemicals. Nanotechnology involves the use of nanomaterials in agriculture to develop methods that limit the use of harmful agrochemicals and help boost the yield of different crops [11]. NPs play a promising role in the treatment of diseases, due to their unique properties [12,13]. However, the morphology, size, and size distribution of the generated NPs have a significant impact on these distinct properties [14]. Selenium nanoparticles (SeNPs) and Cerium oxide nanoparticles (CeONPs) are promisingly superior to other NPs, due to their unique biological, chemical, antibacterial, medical, and pharmacological capabilities [15]. SeNPs and CeONPs are widely used in nanobiotechnology and medical diagnostics because they have higher bioavailability, reduced toxicity, and better absorption capacity than inorganic and organic forms [16,17]. CeONPs have also been used in catalyst systems as metal catalysts, by combining with other metal species and exhibiting anti-inflammatory roles and antimicrobial properties [18].

NPs can be created using a variety of techniques. Currently, most NPs are created using chemicals, which have been observed to be quick, but are toxic, expensive, and unfriendly to the environment [13]. On the other hand, green synthesis, utilizing plant extracts, has gained favor since it is pure, affordable, and biocompatible [19]. In addition, plant-mediated NP synthesis is quicker than that offered by other biological species because there is no need to maintain media and growth conditions [20]. As metal NPs can be made by reducing metal ions, plant extracts may function as reducing agents in the formation of NPs [21]. Reactive oxygen species (ROS), which are produced because of stress, interact with biological elements to impact plant differentiation, development, and metabolism [22]. Plant cells use SOD, POD, flavonoids, and polyphenols to combat the negative effects of ROS [23,24].

Few investigations on the exogenous application of NPs in some plant species have been published thus far. The role of green synthesized SeNPs and CeONPs in terms of antifungal potential, biochemical profiling, and various quality and productivity parameters in wheat under the stress of stripe rust disease is still to be explored. This study was designed to study the effects of green synthesized SeNPs and CeONPs on the physiological, biochemical, and antioxidant parameters of wheat under the stress of stripe rust disease.

## 2. Results and Discussion

### 2.1. Green Synthesis of SeNPs and CeONPs

The green synthesis of SeNPs was carried out using *Melia azedarach* leaves as the main reducing and stabilizing agent. Sodium selenite (Na_2_SeO_3_) stock solution was mixed with *M. azedarach* leaf extract. At the beginning, the reaction mixture was light green in color, but later the color began to change, and, finally, a brick red color was formed which confirmed the formation of SeNPs.

Green synthesis of CeONPs was carried out by using the dry powder of the rhizome of *Acorus calamus*. A stock solution of cerium nitrate Ce(NO_3_)_3_ was prepared, to which the extract was added drop by drop. Initially, the reaction mixture turned into a light brown color, and then precipitation began to settle, which confirmed the synthesis of CeONPs. A certain concentration of active components is thought to be present in plant extract that is important in NP synthesis [25]. The ability to synthesize NPs from plant extracts is important since it reduces downstream processing and avoids the difficulty of maintaining cell cultures [26]. During the reduction reaction, the color of Na_2_SeO_3_ changed from colorless to brick-red, indicative of NP production [27]. The color of Na_2_SeO_3_ solution changed from colorless to brick red in our study, showing that SeNPs were biosynthesized from Na_2_SeO_3_ by *M. azedarach* leaf extract reduction and its stabilizing activities. Our observations were like those of Satgurunathan et al. [28], who used Na_2_SeO_3_ and *Allium sativum* clove extract for the formation of SeNPs.

Plant-based CeONPs are produced through a simple approach, in which bulk metal salt is mixed with the extract and the reaction completes in minutes to a few hours in ordinary laboratory conditions [29]. The metallic salt solution is reduced into respective NPs via the phytochemicals whose synthesis is confirmed firstly through color change from colorless to yellowish, brownish, or whitish [30]. Our results agreed with the previous findings of Yiling et al. [31], who synthesized the CeONPs from the leaf extract of *Azadirachta indica.*

### 2.2. Characterization of SeNPs and CeONPs

**Figure 1:**
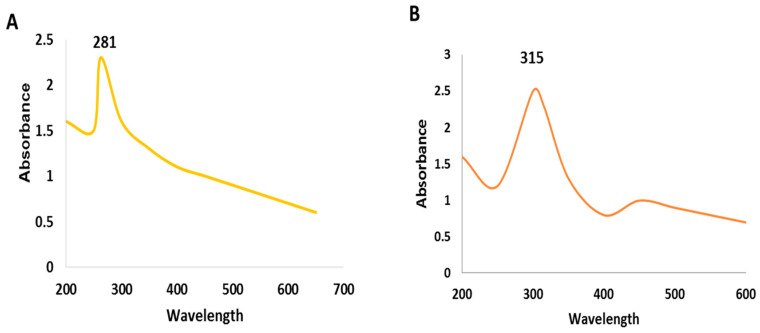
UV–Visible spectrum of the green synthesized nanoparticles (NPs). (**A**) SeNPs, (**B**) CeONPs.

The green synthesized SeNPs and CeONPs were characterized by using UV-Visible spectroscopy, SEM, EDX and XRD.UV-Visible spectroscopy is widely used for reduction and capping of NPs. According to the UV-Visible spectroscopy conducted, the absorbing peak of SeNPs was noted at 281 nm (200–400 nm), while that of CeONPs was at 315 nm (200–400), as shown in Figure 1A,B, which confirmed the formation of SeNPs and CeONPs, respectively. The result of the present study was supported by the previous study of Fesharaki et al. [32], who prepared SeNPs from *Klebsiella pneumonia* that exhibited the highest absorbance at 280 nm. Kokila et al. [33] reported that NPs produced from *Diospyros montana* produced a peak around 261 nm. CeONPs synthesized from *Olea europaea* leaf extract by Maqbool et al. [34], had a maximum absorption peak similar to that observed in our study.

**Figure 2:**
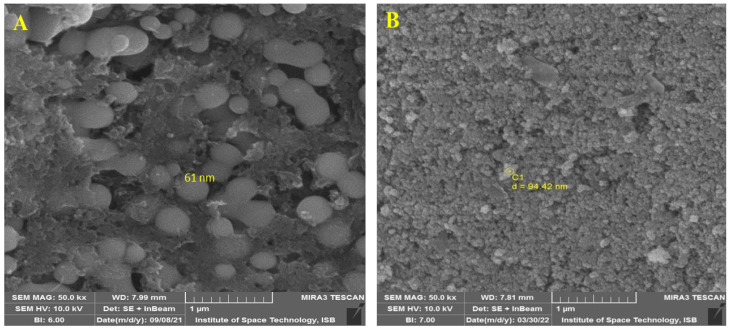
Scanning Electron Microscope (SEM) analysis of the green synthesized nanoparticles (NPs). (**A**) SeNPs, (**B**) CeONPs.

SEM analysis was used to investigate the morphologies of SeNPs and CeONPs and revealed the spherical shape of SeNPs and CeONPs. The average size of SeNPs and CeONPs was approximately 61 nm and 42 nm, respectively (Figure 2A,B). Our study agreed with Verma and Maheshwari [35], who reported a 74.25 nm average size of SeNPs. The results were like those of [33,36,37,38,39], who reported spherical SeNP and CeONP production from plant extracts.

**Figure 3:**
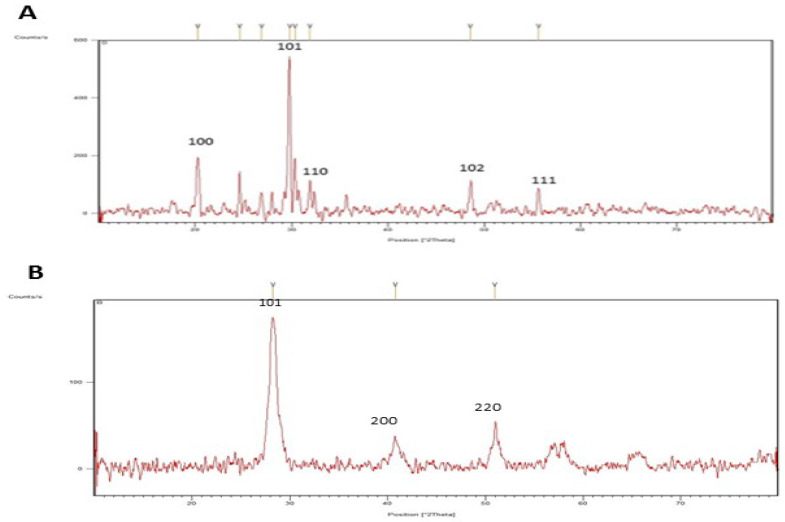
EDX spectrum of the green synthesized nanoparticles (NPs). (**A**) SeNPs, (**B**) CeONPs.

An EDX detector was used to detect the metallic selenium and cerium ions. The sample was analyzed by EDX methods, and energy dispersive microanalysis gave us more insight into the properties of the SeNPs and CeONPs. Based on this bioreduction approach, EDX was used to analyze the elements of the SeNPs and CeONPs. The EDX spectrum showed different absorption peaks of the elements. Different peaks of elements, such as oxygen, together with selenium (Figure 3A), were observed, while different peaks of Ca, K, C, P and S, together with cerium, were detected (Figure 3B). The results showed that the reaction products were in the pure form of SeNPs and CeONPs (Figure 3A,B). These findings agreed with the results obtained from the studies by Fresneda et al. [40], Khan et al. and Alam et al., which all reported the production of crystalline nanostructures [41,42].

**Figure 4:**
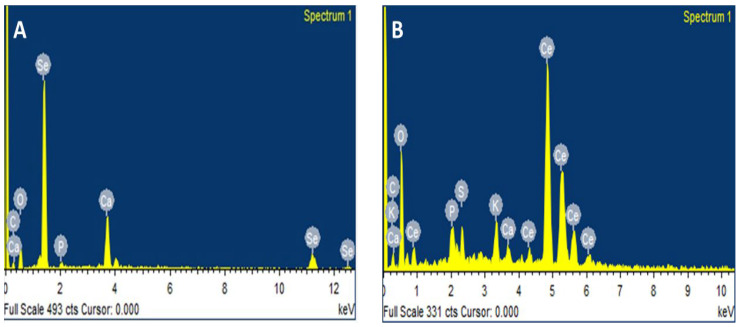
XRD spectrum of the green synthesized nanoparticles (NPs). (**A**) SeNPs, (**B**) CeONPs.

By using the XRD technique, the crystalline structure of bio-fabricated SeNPs and CeONPs was reported. The XRD pattern of bio-fabricated SeNPs exhibited different spectral peaks at 2-theta = 20.25°, 24.593°, 26.862°, 29.809, and 30.327°, that were relevant to their indices and planes of diffractions (100), (110), (101), (111), (102) (Figure 4A). Similarly, the CeONPs indicated diffraction planes (101), (200), (220), (111), and (311) that showed 2-theta angle 28.25°, 40.82° and 50.98°, respectively, as shown in (Figure 4B), and which relate to JCPDS file no: 89-8436 for CeO_2_. The present result agreed with the findings of the previous studies by Rajan et al., 2019 [43]. These diffractions planes were exactly similar to those previously reported by Alamand et al. [44], and Fresneda et al., who reported crystalline SeNPs prepared from plant extract [45].

### 2.3. Evaluation of Disease Severity

**Figure 5:**
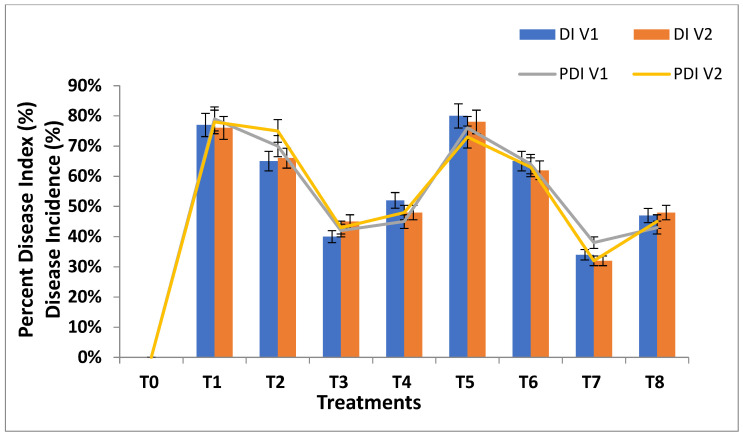
Disease incidence and percent disease index of wheat plants inoculated with *P. striiformis* and treated with green synthesized SeNPs and CeONPs. (Mean ± SE; n = 3). Different small letters on bars statistically significant variation at *p* < 0.05 as per DMRT.

The disease incidence (DI) and the percentage disease index (PDI) of wheat plants were evaluated under *P. striformis* against different concentrations of biosynthesized SeNPs and CeONPs at different time intervals, such as after 5, 10, 15, 20, 25, and 30 days. After 15 days of inoculums and foliar applications of SeNPs and CeONPs, the data was collected to calculate the disease severity. It was found that none of the concentrations of green synthesized SeNPs and CeONPs, could completely prevent stripe rust infection. Nevertheless, treatment with different concentrations of both NPs had a significant effect on disease severity. Over time, there was a steady decline in the incidence of stripe rust in response to all concentrations of green synthesized SeNPs and CeONPs. When wheat plants were infected with stripe rust stress and no NP treatment was applied, disease incidence and disease index were significantly higher. When stripe rust stress was administered at 30 mg/L SeNPs and 30 mg/L CeONPs, the plants were shown to have the lowest disease incidence and disease index. At the concentration of 30 mg/L SeNPs and 30 mg/L CeONPs were applied to yellow rust-affected plants, and the percent disease index values decreased by 60% 66% in (V1) and 78% and 68% in (V2), respectively.Similarly, the percent disease index values of 52% and 57% in (V1), and 62% and 68% in (V2), were calculated after the foliar application of SeNPs and CeONPs (Figure 5). The concentration of 30 mg/L of biosynthesized SeNPs and CeONPs caused a significant decrease in disease incidence and percent disease index under stripe rust disease stress at different time intervals. Similar results were previously reported by Iqbal et al. [46].

### 2.4. Effect of Exogenous Application of SeNPs and CeONPs on Morphological Profiles of Wheat

**Figure 6:**
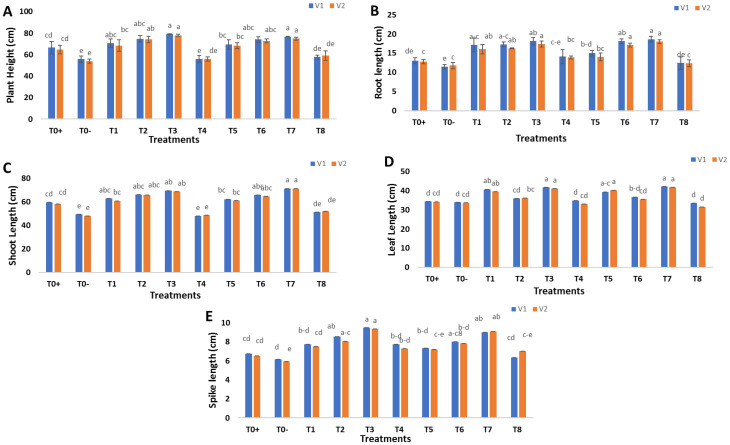
Effect of foliar applications of green synthesized SeNPs and CeONPs on morphological parameters of wheat plant under the stress of stripe rust disease. To+ represent the control, To− represents the effect of pathogen only, T1 to T4 (i.e., 10, 20, 30, 40 mg/L) represents the effect of green synthesized SeNPs, T5 to T8 (i.e., 10, 20, 30, 40 mg/L) represents the effect of green synthesized CeONPs (**A**) Plant height in response of green synthesized SeNPs and CeONPs (**B**) Root length in response of green synthesized SeNPs and CeONPs. (**C**) Shoot length in response of green synthesized SeNPs and CeONPs. (**D**) Leaf length in response of green synthesized SeNPs and CeONPs. (**E**) Spike length in response of green synthesized SeNPs and CeONPs. (Mean ± SE; n = 3). Different small letters on bars represent statistically significant variation at *p* < 0.05 as per DMRT.

The morphological parameters of wheat were investigated to analyze the potential of green synthesized SeNPs and CeONPs against stripe rust disease. Different concentrations of SeNPs and CeONPs (see Table 1) were applied as a foliar spray on the two varieties of wheat and morphological parameters were recorded in terms of plant height, root length, shoot length, leaf length, and ear length. Initially, a reduction in growth characteristics was observed in both wheat varieties. After foliar spraying with SeNPs and CeONPs, these parameters significantly improved at all concentrations, but the most significant results were recorded at a concentration of 30 mg/L of both SeNPs and CeONPs (Figure 6). The findings of the present study were in accordance with previous studies. Siddiqui et al. [47] reported that *Hordeum volgare* morphological characteristics were improved by foliar spray of SeNPs. A similar investigation was carried out by Desoky et al. [48]. The growth characteristics obtained by foliar spray of CeONPs were identical to those obtained by foliar spray of CeONPs [49]. Rico et al. [50] used CeONPs to analyze the growth characteristics of *H. vulgare* L. in an experiment. Similarly, the treatment of CeONPs to *Zea mays* L. resulted in enhanced growth characteristics [51].

### 2.5. Effect of Exogenous Application of SeNPs and CeONPs on Physiological Profiles of Wheat

**Figure 7:**
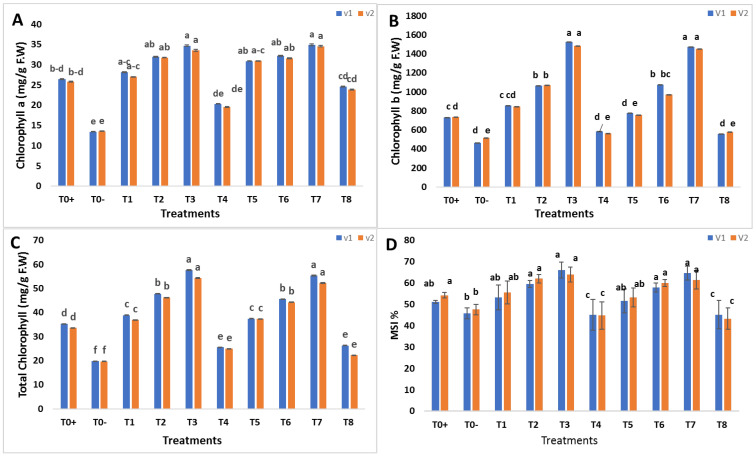
Effect of foliar applications of green synthesized SeNPs and CeONPs on physiological parameters of wheat plant under the stress of stripe rust disease. To+ represent the control, To− represents the effect of pathogen only, T1 to T4 (i.e., 10, 20, 30, 40 mg/L) represents the effect of green synthesized SeNPs, T5 to T8 (i.e., 10, 20, 30, 40 mg/L) represents the effect of green synthesized CeONPs (**A**) chlorophyll a content in the response of green synthesized SeNPs and CeONPs (**B**) chlorophyll b content in the response of green synthesized SeNPs and CeONPs. (**C**) Total chlorophyll contents in the response of green synthesized SeNPs and CeONPs. (**D**) Membrane stability index (MSI) in the response of green synthesized SeNPs and CeONPs. (Mean ± SE; n = 3). Different small letters on bars represent statistically significant variation at *p*< 0.05 as per DMRT.

The physiological parameters, such as chlorophyll and membrane stability index, of wheat plants were assessed to evaluate the effects of biosynthesized SeNPs and CeONPs under stripe rust disease. The overall result reported that all treatments with both NPs worked well, and SeNPs T3 = 30 mg/L and CeONPs T7 = 30 mg/L proved to be the most effective concentrations for increasing photosynthetic pigments and membrane stability index (Figure 7A–D). Chlorophyll a and b are essential pigments in the process of photosynthesis, which consists of two processes. The light reactions, in which NADPH and ATP are produced, and the dark reaction, in which carbon dioxide is fixed, are two examples of such reactions [52]. The increase in chlorophyll content caused by the application of various NPS could be due to the increased water uptake by plants, according to Khan et al. [53]. Another likely explanation is that NPs reduce oxidative stress, allowing plants to enhance their photosynthetic process [54,55]. The results of the present study were supported by a previous study by Du et al. [56], who used CeONPs to improve physiological and biochemical properties of wheat. Similarly, Rico et al. [50], reported that CeONPs improved the physiological parameters of *Hordeum vulgare* L. The foliar spray of CeONPs also increased the total chlorophyll content in sorghum [57]. The findings of this study were like those of Quiterio-Gutiérrez et al. [58]. Zahedi et al. [59], reported that SeNPs improved the chlorophyll content of tomato plants under *Alternaria solani* stress. Dong et al. [60], found that SeNPs increased the chlorophyll content in *Lyciumchinense* leaves by 200–400%. The present results agreed with those of Rady et al. [61], who reported that SeNPs promoted physiological attributes against *Phaseolus vulgaris*. 

The increase in MSI of the present study were in agreement with the previous studies by Desoky et al. [61], Fox et al. [51], and Mohammadi-Cheraghabadi et al. [62]. As a result, an increase in chlorophyll and MSI in stripe rust-infected plants treated with SeNPs and CeONPs could help restore photosynthetic machinery and, hence, growth qualities.

### 2.6. Exogenous Application of SeNPs and CeONPs on Biochemical Attributes of Wheat

**Figure 8:**
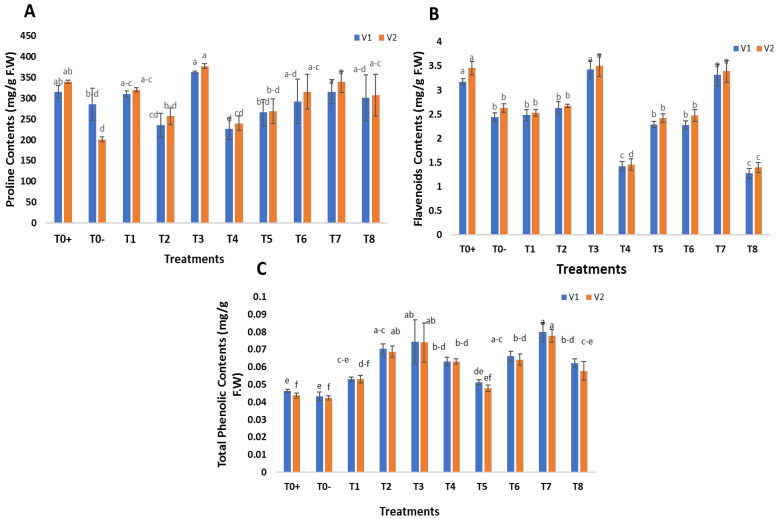
Effect of foliar applications of green synthesized SeNPs and CeONPs on biochemical parameters of wheat plant under the stress of stripe rust disease. To+ represent the control, To− represents the effect of pathogen only, T1 to T4 (i.e., 10, 20, 30, 40 mg/L) represent the effect of green synthesized SeNPs, T5 to T8 (i.e., 10, 20, 30, 40 mg/L) represent the effect of green synthesized CeONPs (**A**) Proline Contents, (**B**) Total Flavonoids Contents (**C**) Total Phenolic Contents. (Mean ± SE; n = 3). Different small letters on bars represent statistically significant variation at *p* < 0.05 as per DMRT.

The effects of foliar application of green synthesized SeNPs and CeONPs on biochemical parameters were recorded in terms of proline, phenolics and flavonoids. The proline, phenolics and flavonoid contents were analyzed in untreated diseased plants and treated with different concentrations of SeNPs and CeONPs. The biochemical parameters were enhanced by the foliar applications of green synthesized SeNPs and CeONPs. The most significant results were obtained at 30 mg/L of both SeNPs and CeONPs, which most enhanced the proline, phenolic and flavonoid contents (Figure 8A–C).

Proline is an amino acid with multiple functions that helps in defense against plant pathogens [63]. Proline is believed to maintain osmolality, scavenge ROS, stabilize subcellular membranes and proteins, and buffer cellular redox potential in stress situations [64]. Phenolic and flavonoid molecules play a crucial role in protecting plants from the negative effects of various stresses. The results of Ghasemian et al. [65] supported our findings on the use of SeNPs in foliar spray to determine total proline content. Sardar et al. [66] reported that plants raised from seeds primed with SeNPs exhibited enhanced proline and soluble sugar contents. The current study supported the findings of prior research [24,61,67]. The results of the present study were in agreement with those of Jahani et al. [68], who used CeONPs to improve physiological and biochemical parameters in *Calendula officinalis* L., and the study by Quiterio-Gutierrez et al.’s on mung beans [58].

According to Raigond et al. [69], zinc NPs promoted the concentration of phenolic compounds in potato plants, which accorded with our results. Similarly, the current results confirmed those of Lopez-Vargas et al. [70], who discovered that CuNPs increased flavonoids in tomatoes by 36.14 percent.

### 2.7. Foliar Spray of Green Synthesized SeNPs and CeONPs on Antioxidant Defense System of Wheat

**Figure 9:**
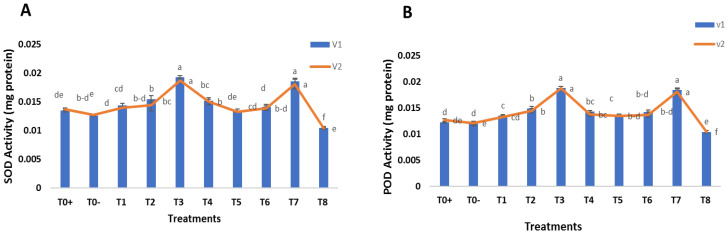
Effect of foliar applications of green synthesized SeNPs and CeONPs on antioxidant defense system of wheat plant under the stress of stripe rust disease. To+ represent the control, To− represents the effect of pathogen only, T1 to T4 (i.e., 10, 20, 30, 40 mg/L) represent the effect of green synthesized SeNPs, T5 to T8 (i.e., 10, 20, 30, 40 mg/L) represent the effect of green synthesized CeONPs (**A**) SOD Activity and (**B**) POD Activity. (Mean ± SE; n = 3). Different small letters on bars represent statistically significant variation at *p* < 0.05 as per DMRT.

The overall result reported that antioxidant enzymes, such as superoxide dismutase (SOD) and peroxidase (POD), increased in both wheat varieties under disease stress by foliar application of SeNPs and CeONPs. In the positive and negative control, SOD and POD were detected at low levels. The most significant results were obtained at 30 mg/L of both SeNPs and CeONPs, which most enhanced the SOD and POD (Figure 9A,B). Antioxidant enzymes and molecular chaperones are important proteins that play essential roles in protective mechanisms [70]. One of the protective mechanisms of the enzymatic antioxidant system is the enzyme SOD, which is found in various areas of the cell. The dismutation of superoxide radicals into hydrogen peroxide and oxygen is catalyzed by SOD. Since plant superoxide dismutase (SOD) catalyzes the removal of the free radical O_2_, a decrease in the activity of SOD leads to an accumulation of the free radical O_2_ in leaves [71]. 

The findings of this work were consistent with those of previous studies. SeNPs increased the level of SOD in stressed sorghum plants, resulting in greater stress tolerance in [57]. Similarly, several additional investigations found that SeNPs increased POD and SOD activities in stressed strawberry plants [59]. Jiang et al. [72], found that selenium treatments triggered antioxidant defense genes, and enhanced the content of SOD, in corn, resulting in higher stress tolerance in plants. Our results agreed with those of Raigond et al. [69], in which metal NPs increased antioxidant enzymes, such as POX and APX, in potato plants. The increase of POX and APX could be an important factor in the decomposition of H_2_O_2_, especially in the absence of CAT. Similarly, CeONPs increased the activity of SOD in mung beans in the study by Kamali-Andani et al. [73].

### 2.8. Antioxidant Activity of SeNPs and CeONPs

**Figure 10:**
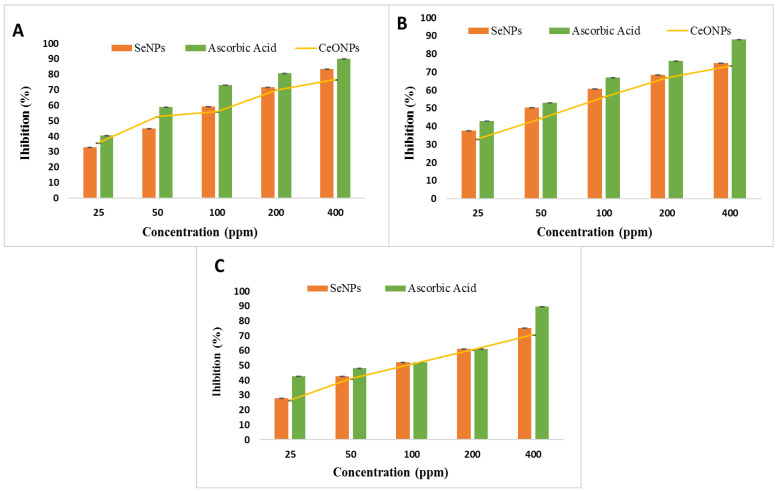
Effect of foliar applications of SeNPs and CeONPs on Antioxidant Activity (**A**) DPPH Assay. (**B**) ABTS Assay, (**C**) Reducing Power Assay of SeNPs and CeONPs.

The antioxidant activity of SeNPs and CeONPs was measured using the DPPH and ABTS and reducing power assays. The SeNPs and CeONPs showed strong DPPH, ABTS and reducing power radical scavenging activity, which was dose-dependent (Figure 10A–C). This indicated that the antioxidant activity was proportional to the concentration of SeNPs and CeONPs. Both SeNPs and CeONPs showed a significant increase at 400 ppm. SeNPs had a DPPH value of 83.33 percent, an ABT value of 74.84 percent and reducing power value of 75.24 percent, while CeONPs had a DPPH value of 76.66 percent, an ABT value of 73.36 percent and reducing power value of 70.6 percent. The standard values were reported as 90 percent DPPH value and 87.84 percent ABT value. Our results were similar when compared with previous reports on green synthesized SeNPs. Kokila et al. [33] recently discovered that green synthesized SeNPs, with a size of 16 nm, had an EC50 value of 22.5 g/m. Different concentrations of SeNPs and CeONPs (50, 100, 150, 200, and 400 ppm) were used to test the effectiveness of the reducing power. The reducing power was enhanced by increasing the concentrations of both NPs. Both NPs functioned effectively at 400 ppm, with 75.24 percent of SeNPs and 70.63 percent of CeONPs showing a reducing effect. Cao et al. [74] investigated the reducing effect of SeNPs and found similar results. Small SeNPs exhibited a large specific surface area, which allowed many reactive sites for free radicals. As a result, ultrasonic cavitation contributed to the ability to scavenge free radicals in the study by Khai et al. [75]. In addition, Khai et al. [76], reported that SeNPs had a positive effect on free radical scavenging activity.

## 3. Materials and Methods

### 3.1. Phyto- Synthesis of SeNPs and CeONPs

The green synthesis of SeNPs were performed by following the methodology of Satgurunathan et al. [28]. Sodium selenite (Na_2_SeO_3_) (SIGMA) is a popular salt used in the manufacture of SeNPs. A Na_2_SeO_3_ (10 mM) solution was prepared by dissolving 1.25 g of Na_2_SeO_3_ in 500 mL of distilled water and heating at 80 °C for 30 min with magnetic stirring on a hot plate (Sr # G150). To reduce sodium salt to SeNPs, *Melia azedarach* leaves extract was used. The *M. azedarach* extract was made by boiling the leaves (4.69 g) in 100 mL distilled water for 5 min, according to the methodology of Fardsadegh [76]. The *M. Azedarach* extract was gradually added to the Na_2_SeO_3_ solution while it was continuously boiled at 100 °C until a brick red hue developed. The solution was subjected to centrifugation for 15 min at 25 °C at 10,000 rpm. A Speed Vac concentrator was used to dry the pellet. The generated SeNPs were then characterized, before being utilized as a foliar spray on wheat plants to combat the stripe rust disease.

The rhizome of *Acorus calamus* was used for the synthesis of CeONPs. For the preparation of the rhizome extract, fifty grams of rhizome powder was added to 500 mL double distilled water and heated for 15 min at 100 °C. After cooling to room temperature, the mixture was filtered through Whatman filter paper. To obtain a clean, particle free extract solution, the filtrate was centrifuged at 12,000 rpm for 20 min. The prepared extract was immediately used for the synthesis of CeONPs by following the protocol of Altaf et al. [37] with minor modifications. For this, 10 mL of plant extract was poured into 100 mL cerium (III) nitrate stock solution (100 mM), which was agitated for 4 h using a magnetic stirrer. The solution turned light brown, and the precipitate began to settle. The reaction mixture was centrifuged at 15,000 rpm for 30 min, before being rinsed with ethanol and centrifuged again. The residue was dried completely in a vacuum oven at 80 °C, before being pulverized with a mortar and pestle. The powder was then calcined at 400 °C for 2 h. Finally, the powder was ground to a fine powder and stored at room temperature until use.

### 3.2. Characterization of Nanoparticles

#### 3.2.1. UV-Visible Analysis of SeNPs and CeONPs

The UV-Visible optical absorption properties were measured using a spectrophotometer (Hermal Germany model Z326k, Reinbek, Germany). The absorption spectrum was measured in the wavelength range of 200–800 nm. Early characterization of CeONPs was performed by UV-Visible spectroscopy. To prepare a homogeneous suspension, the powdered NP sample was suspended in distilled water. The UV-Visible absorption spectrum of CeONPs was measured from 250 to 500 nm using a Shimadzu UV-2600 spectrophotometer. The absorbance was measured at room temperature using a blank sample of double distilled water.

#### 3.2.2. Scanning Electron Microscopy of SeNPs and CeONPs

A scanning electron microscope (SEM) was used to study the morphology and size of SeNPs and CeONPs. Images of the samples were acquired using a conventional secondary electron detector and a 10-kV electron beam. The NP powder was placed on double-sided tape with one side glued to the sample holder and the other to the sample. Under vacuum, the samples were then sputtered with a thin layer of gold.

#### 3.2.3. Energy Dispersive X-ray (EDX)

Elemental analysis of green synthesized SeNPs and CeONPs was also performed at IST, Islamabad, using EDX detector (SIGMA model).

#### 3.2.4. X-ray Diffraction (XRD)

The crystalline nature of the green synthesized SeNPs and CeONPs was determined using X-ray diffraction (XRD) at the NCP, Islamabad. It was done by placing a powdered sample of SeNPs and CeONPs on Shimadzu XRD-6000, set in the range of 5–50° at a 2θ angle [77,78,79]. Average size of NPs was determined by applying Debye–Scherer’s equation:

D = Kλ/βcosθ

where K = shape factor, λ = X-ray wavelength, β = full width in radius at half maximum and θ = Bragg’s angle.

### 3.3. Glass House Experiment

In a glass house experiment, the antioxidant and antifungal activity of greens ynthesized SeNPs and CeONPs in wheat against *P. striformis* were investigated. Several earthen pots, with a capacity of nearly 10 kg, were filled with sterilized soil. The soil texture was sandy loam with silt (20%), clay (35%) and sand (25%) as the main components (40%). Seeds of disease susceptible wheat varieties, Galaxie-13 (V1) and NARC 2011 (V2), were obtained from NARC Islamabad. Sufficient surface sterilization of wheat seeds was achieved with 0.1% mercuric chloride. Sowing was completed on 25 October 2021. No more than five seedlings were kept in each pot. Experiments were conducted using a completely randomized design (CRD), with three replicates for each treatment. The experiments were first conducted with low concentrations of SeNPs and CeONPs. Then, concentrations were selected for foliar spraying to evaluate potential effects on rust fungal growth, compared to control plants, in the current greenhouse experiment. The general experimental design is described in Table 1.

### 3.4. Inoculums Preparation

For the preparation of the inoculums, *P. striformis* strain (accession No. 572432) was obtained from the Crop Disease Research Institute of the NARC, Islamabad, Pakistan. The spore suspension was prepared by suspending the uredinio sporesin distilled water, and 1 mg/L Tween 20 was added for surfactant and the number of spores were counted with the help of a hemocytometer. For this, 0.6 mL of spore suspension was prepared at the rate of 6 × 10^5^ spores /mL to confirm the required spore concentration, and 20 µL of inoculum was placed on a grid. The spherical spores with a bright yellow color were further used [11].

### 3.5. Inoculation of Fungus and Foliar Application of SeNPs and CeONPs on Wheat

The spore suspension of *P. striformis* was sprayed directly onto the leaves of wheat plants at the flag leaf stage, using an atomizer. The sprayed suspension had a volume of 60 mL per plant. After inoculation, wheat plants were sprayed with distilled water and covered with transparent polyethylene bags to maintain 95–100% humidity at a temperature of 15–18 °C. Plants were also covered with plastic sheets to prevent spore transmission. Plants were sprayed with different concentrations (10, 20, 30, and 40 mg/L SeNPs and 10, 20, 30, 40 mg/L CeONPs) along with inoculums. Initial data were collected one week after disease inoculation and every week thereafter.

### 3.6. Collection of Samples for Disease Severity

The leaf tissues for the analysis were taken at random from the triplicate. The intensity of stripe rust symptoms was evaluated using a rating scale based on visual observation (Table 2).

The symptoms revealing the severity of stripe rust were assessed by the use of a rating scale on a visual basis (Table 2), as described by Iqbal et al. [46].

The estimation of disease incidence was determined by through the methodology of Iftikhar et al. [49], using Equation (1):

(1) $$Disease\;incidence\;\left(\%\right)=\frac{Number\;of\;rust\;infected\;plants}{Sum\;of\;all\;plants}\times 100$$

The disease severity in percentage is called the disease index and can be calculated by using the methodology of Iftikhar et al. [49], as shown in Equations (2) and (3):

(2) $$Percent\;disease\;index=\frac{Disease\;Index}{Total\;Infected\;Plants}\times 100/5$$

Disease index = (Stripes in scale one) + (Stripes in scale two) + (Stripes in scale five)(3)

### 3.7. Evaluation of Plant Morphological Parameters

The wheat samples were collected from each treatment and root, shoot, leaf, and spike were separated to measure the length of the growth parameters using an ordinary measuring scale. Similarly, whole plants were uprooted to measure plant height.

### 3.8. Evaluation of Plant Physiological Parameter

#### 3.8.1. Chlorophyll Contents (mg/g F.W)

Chlorophyll contents of the leaf were measured by using a spectrophotometer (Model U-2900 Sr. No 26E82-018). Plant leaves, each weighing 2 g after washing were ground in 10 mL of acetone. After grinding, the solutions were filtered into other test tubes in one more set of test tubes, and absorbance was measured at 645 nm, 652 nm, and 663 nm wavelength [80]. The subsequent equations used to calculate the chlorophyll contents of the leaves were:

Cha = 12.7(A663) − 2.7(A645)

Chb = 22.9(A645) − 4.7(A663)

Chl_total_ = (A652 × 1000/34.5)

#### 3.8.2. Membrane Stability Index (%)

The MSI% was measured using the protocol of Sairam et al. [81]. The 100 mg discs of leaves taken from each treatment were put into test tubes and test tubes were placed in a water bath for 30 min at 40 °C. The electrical conductivity (C1) was then measured using an EC meter. The electrical conductivity of the test tubes (C2) was measured after 10 min in a water bath at 100 °C. The membrane stability index was calculated using the formula:

$$MSI=1-\frac{C1}{C2}\times 100$$

### 3.9. Evaluation of Plant Biochemical Parameters

#### 3.9.1. Proline Contents

By using the ninhydrin method, the Proline contents of leaves were estimated by following the methodology described by Khan et al. [82]. Fresh leaves of weight 0.5 g were crushed, using a pestle and mortar, and mixed with sulfosalicylic acid (3%). The 2 mL plant extract, 2 mL of ninhydrin reagent and 2 mL of glacial acetic acid were homogenized. At 100 °C the resulting mixture was boiled in a water bath for 30 min and, then, 6 mL toluene was poured into the sample after cooling and transferred to a separating funnel to obtain the layer. The absorbance was measured at 520 nm using a spectrophotometer.

#### 3.9.2. Total Flavenoid Contents

Plant extract and 0.9 mL Folin–Ciocalteau reagent (10%) were added to the reaction mixture, and, then, 0.6 mL sodium carbonate (7.5 percent, *w*/*v*) solution was added. The solution was allowed to sit at room temperature for 1 h before the absorbance was recorded at 760 nm. Then, in the range of 100–600 ppm, a known concentration of Gallic acid aqueous solution was used for calibration. The results were represented in milligrams of Gallic acid equivalents (GAE) per gram of fresh weight (FW), in accordance with Hussain et al. [24].

#### 3.9.3. Total Phenolic Contents

The total phenolic contents of wheat were assayed by using the protocol described by Ashraf et al. [83]. The methanolic extracts (1 mL) with 5 mL distilled water were placed in clean test tubes. After that, 50% Folin–Ciocaltu reagent was added into the samples and incubated for 30 min in dark conditions and, then, 1 mL of 50% sodium carbonate was added and again the samples were incubated for 10 mints in the dark. The absorbance was measured at 725 nm, using a spectrophotometer.

#### 3.9.4. SOD Activity

The SOD Activity was assayed according to Joshi et al. [84]. A fresh leaf sample of weight 1 g was homogenized with 5 mL of the 50 milli molar potassium phosphate buffer (pH 7.4), using a clean pestle and mortar, and the resulting mixture was subjected to centrifugation at 12,000 rpm for ten minutes at 4 °C. The supernatant was used as enzyme extract and 500 μL enzyme extract +13 mM methionine +75 μM NBT+ 2 Um riboflavin + 0.1 milli molar EDTA were mixed. Before shaking under bright light for 20 min, riboflavin was mixed in and the test tubes were covered with aluminum foil. The absorbance was taken at 560 nm using a UV-Visible spectrophotometer.

#### 3.9.5. POD Activity

The POD activity was assayed by using the protocol followed by Joshi et al. [84]. The reaction mixture consisted of 1 mL of 20 times diluted enzyme extract, 125 μM phosphate buffer pH 6.8, 50 μM hydrogen peroxide, and 50 μM pyrogallol. The resulting color was due to the amount of purpurogallin and was measured at the absorbance of 420 nm using a spectrophotometer.

### 3.10. Antioxidant Activity

#### 3.10.1. DPPH Assay

Various concentrations of SeNPs and CeONPs (25, 50, 100, 200, 400 ppm) were mixed with 0.5 mL of DPPH solution (250 M in methanol) + 1 mL of 0.1 M acetate buffer in a DPPH radical scavenging test, and the total volume was brought up to 3 mL with methanol. The reaction mixture was carefully mixed before being kept in the dark for 30 min at 27.2 °C. A UV-Visible spectrophotometer was used to detect the absorbance at 517 nm. Ascorbic acid was utilized as a standard, and the reaction mixture without SeNPs and CeONPs as a control, in accordance with Gunti et al. [85]:

DPPH (%) = (1 − ATS) AC × 100

where, ATS and AC were absorbance of the test sample and control, respectively.

#### 3.10.2. ABTS Assay

For the ABTS radical scavenging assay, an ABTS radical solution was prepared by reacting 7 mM ABTS with 2.45 mM potassium persulfate (1:1) in water and incubating for 12 h at 27.2 °C in the dark. By diluting the ABTS radical solution with methanol, the optical density at 734 nm was adjusted to 0.7. Then, different concentrations of SeNPs and CeONPs (25, 50, 100, 200, 400 ppm) were added to 3 mL of the ABTS solution (0.7 optical densities) and incubated for 6 min at 27 °C in the dark, and the optical density at 734 nm was measured with a spectrophotometer. The ABTS solution without the test sample was used as a control and ascorbic acid was used as a standard, in accordance with Gunti et al. [85]. This activity was measured through the given formula:

ABTS radical Assay (%) = (1 − ATS) AC × 100

where, ATS and AC were absorbance of the test sample and control, respectively.

#### 3.10.3. Reducing Power Assay

The capacity of SeNPs and CeONPs to scavenge ABTS radical cations was investigated. By mixing 7.4 mmol/L ABTS solution with 2.6 mmol/L potassium persulphate, the ABTS radical cation (ABTS percent+) was created. After being kept in the dark for 12–16 h at room temperature, the ABTS % + solution was diluted to a stable absorbance of 0.70 0.01 at 734 nm. Then, at different concentrations (0.33–1.67 mg/mL), 1.4 mL of the sample solution was mixed with 0.7 mL of diluted ABTS percent + solution. After 6 min of reaction at room temperature, the absorbance was measured with a spectrophotometer at 734 nm. Ascorbic acid was used as standard, in accordance with Cao et al. [74]. The ability of SeNPs and CeONPs to scavenge ABTS% + was calculated using the following formula:

ABTS % + scavenging ability (%) = [1 − (A1 − A2)/A0] × 100

### 3.11. Statistical Analysis

The experiment was replicated thrice, and measured data were statistically analyzed using Statistics 8.1. To test the overall significance of the data, ANOVA (analysis of variance) was calculated using least significant difference (LSD) at a 5% probability level for comparison of means.

## 4. Conclusions

The results of the present study explained the effect of SeNPs and CeONPs on wheat plants under the stress of stripe, or yellow rust, disease. SeNPs and CeONPs reduced the disease index % of stripe, or yellow rust, disease of wheat plants. It was reported that SeNPs and CeONPs with a concentration of 30 mg/L significantly improved the morphological (i.e., plant height, root length, shoot length, leaf length, and spike length and physiological (i.e., photosynthetic pigments and membrane stability index) parameters. Total proline, phenolic and flavonoid contents were also increased, compared to control. Similarly, the plant’s defense system, in terms of SOD and POD activity, was significantly improved. The antioxidant activity of SeNPs and CeONPs, in terms of the DPPH assay, the ABTS assay, and the reducing power assay, were also improved. There is a need to increase usage of NPs to control wheat diseases, especially stripe rust disease. Biogenic SeNPs and CeONPs are widely expected to be efficient and cost-effective treatments for fungal plant diseases. Before commercial usage in plant disease control in the field, the adverse effects of these biogenic NPs on agriculture and ecosystems should be determined.

## Figures and Tables

**Table 1:** Treatment layout for evaluation of disease severity and SeNP and CeONP concentrations.

Treatments	Concentrations (mg/L)
To (Positive)	Control (healthy wheat plants)
To (Negative)	Pathogen (*P. striiformis)*
T1	10 mg/L of SeNPs + Pathogen
T2	20 mg/L of SeNPs + Pathogen
T3	30 mg/L of SeNPs + Pathogen
T4	40 mg/L of SeNPs + Pathogen
T5	10 mg/L of CeONPs + Pathogen
T6	20 mg/L of CeONPs + Pathogen
T7	30 mg/L of CeONPs + Pathogen
T8	40 mg/L of CeONPs + Pathogen

**Table 2:** Rating scale for yellow or stripe rust disease.

0	No Symptoms	Resistant
1	1–5% stripes on the leaves	Moderately resistant
2	6–20% stripes on the leave	Moderately resistant
3	21–40% stripes on the leaves	Moderately susceptible
4	41–60% stripes on the leaves	Moderately susceptible
5	>61% stripes on the leaves	Susceptible

## Data Availability

All the obtained data are presented in this article.
